# Preference for primary care in Chinese homebound patients

**DOI:** 10.1186/s12889-024-17910-6

**Published:** 2024-02-12

**Authors:** Jinxin Zhang, Xiaojie Sun, Aaron Yao

**Affiliations:** 1 Centre for Health Management and Policy Research, School of Public Health, Cheeloo College of Medicine, Shandong University, 44 Wenhuaxi Rd, 250012 Jinan, Shandong China; 2 NHC Key Lab of Health Economics and Policy Research, Shandong University, 250012 Jinan, China; 3 Home Centered Care Institute, Schaumburg, IL USA; 4 University of Virginia, Charlottesville, VA USA

**Keywords:** Primary healthcare, Patient preference, Homebound Person, China

## Abstract

**Objective:**

This study aims to describe the preference for primary healthcare (PHC) and investigate associated factors among homebound residents in both rural and urban areas of China. It provides valuable insights to facilitate the rational allocation of healthcare resources and promote the utilization of PHC.

**Methods:**

In this nationally representative cross-sectional study, we utilized the most recent data (2020) from the China Family Panel Studies (CFPS). Participants were recruited from 25 provincial-level administrative regions in both rural and urban areas of China. Homebound patients were asked to provide details about their individual characteristics, variables related to family caregiving, and preferences for PHC. Multivariable logistic models were used to analyze potential factors associated with preference for PHC. Estimates of association were reported as odds ratios (OR) and their 95% confidence intervals (CI).

**Results:**

The study found that 58.43% of rural patients reported a preference for PHC, while 42.78% of urban patients favored PHC. Compared to rural participants who did not received inpatient care in the past year, those who received inpatient care in the past year had 67% lower odds of choosing PHC (OR:0.33, 95% CI:0.19–0.59); Compared to rural participants who did not received family caregiving when ill, those who received family caregiving when ill had 59% lower odds of choosing PHC (OR: 0.41, 95% CI:0.21–0.77). Correspondingly, Compared to urban participants who did not received inpatient care in the past year, those who had received inpatient care in the past year had 75% lower odds of choosing PHC (OR: 0.25, 95% CI: 0.10–0.56); Compared to urban participants who did not received family caregiving when ill, those who received family caregiving when ill had 73% lower odds of choosing PHC (OR: 0.27, 95% CI: 0.11–0.63); Compared to urban participants who with agricultural Hukou, those with Non-agricultural Hukou had 61% lower odds of choosing PHC (OR: 0.39, 95% CI:0.18–0.83); Compared to urban participants living in the eastern part of mainland China, those living in the central part of China had 188% higher odds of choosing PHC (OR: 2.88, 95% CI: 1.14–7.29).

**Conclusion:**

Policymakers should focus on tailoring PHC to vulnerable populations and prioritizing family-based public health strategies for enhancing homebound patients’ perceptions of PHC. Furthermore, further study is needed on whether the Hukou registration system affects the barriers that homebound patients experience in choosing healthcare providers.

## Introduction

The number of homebound patients is increasing rapidly due to the growing population of adults with multimorbidities and functional limitations [1]. A homebound status is defined by Inoue and Matsumoto in 2011 as a community-dwelling person who is unable to leave home without the assistance of others for any reason (e.g., health conditions, functional disability, etc.), and there is no limit to the personal reasons for leaving home [2]. Homebound patients require assistance to address their health and social problems [3]. In the United States, the prevalence of homebound populations is 5.6% [4], and the number of homebound adults has steeply increased during the COVID-19 pandemic. In China, the sharp increase in the number of homebound patients is largely due to the growing number of functionally limited adults who require assistance with basic daily activities [5]. In 2020, the prevalence of homebound populations aged 45 years old in China was 6.2% [6].

Compared to non-homebound patients, homebound patients’ healthcare services utilization is higher [4], but they often delay seeking care [7]. Some well-established models of formal care practice that offer an opportunity to reach high-risk, high-need patients exist, such as the CAPABLE and PACE programs in the United States, the PRISMA program in Canada, and Integrated Care Systems in England [7–8]. These programs emphasize an integrated hospital-community-home care model for adults with limited mobility to support homebound patients’ use of healthcare services [9]. Since 2015, the Chinese government has been expanding medical services to communities and homes for specific groups, such as older patients with chronic diseases and individuals with mental illnesses [10]. Primary healthcare (PHC) institutions in China have implemented various home visit service models, including the family doctor program and home beds, which involve regular visits by designated healthcare professionals who provide treatment and care while documenting the process in medical records. Additionally, initiatives like the national basic public health service project and long-term care insurance have also supported home visits for specific groups, providing preventive care, rehabilitative services, medications, and hospice care [11].

Unfortunately, healthcare institutions in economically developed urban areas (e.g., Beijing, Shanghai) regionally provide only limited door-to-door medical services and nursing services to patients with mobility disabilities near the healthcare institutions [10–11]. Homebound patients who live in socioeconomically disadvantaged areas face disproportionately higher challenges in accessing home visit services [10–11]. Furthermore, in China, there is currently no national long-term care (LTC) insurance system to pay for home visit services for homebound adults; thus, most homebound patients typically seek healthcare by going to healthcare organizations themselves. Additionally, China’s healthcare system currently does not have a strict referral system, and the choice of different medical institutions is based on the healthcare seekers’ preferences [12].

China has a well-established healthcare delivery system consisting of three tiers: primary, secondary, and tertiary healthcare institutions, which provide health interventions for residents. Secondary and tertiary hospitals specialize in advanced diagnostics and treatments for complex diseases, primarily offering inpatient services for specialized care. In contrast, primary healthcare (PHC) institutions, such as community health service centers/stations, township health centers, and village clinics, serve as the most effective and cost-efficient platforms for essential disease diagnosis, treatment, and long-term chronic disease management [12]. PHC is also the foundation for implementing Universal Health Coverage and achieving equity [13]. Importantly, the choice of PHC is a decision of utmost significance for patients in terms of their overall health and well-being [14].

Since the healthcare reform in 2009, the Chinese government has been committed to strengthening its PHC system by improving infrastructure, increasing financial subsidies, enhancing diagnosis and treatment capabilities, and implementing a series of beneficial health policies, such as promoting hierarchical diagnosis and treatment and differentiation of medical insurance reimbursement [12–13]. Studies have shown that patients who receive PHC have greater access to services, earlier management of health problems, a greater focus on prevention, better quality of care, and lower mortality rates compared to those who do not seek PHC [15]. Importantly, PHC provides a pathway for accessing generalist, convenient, and effective health interventions at an affordable cost throughout the life course, including periods of homebound status [14–15]. Middle-aged adults, aged 45 to 65, undergo a transition to old age. This period is marked by significant life changes, including the presence of two or more long-term health conditions, shifts in labor income, and the approach or onset of retirement [16]. Older adults, aged 65 years and above, often face an elevated risk of health issues [16]. Exploring preferences for primary care among the homebound population aged 45 years and older can provide valuable insights to guide health policy decisions. However, it is not clear how homebound patients respond to various choices for PHC.

To our knowledge, the current literature on PHC preferences among individuals aged 45 and above show a preference rate of 62.8% [17]. However, due to the unique circumstances of homebound populations, general population findings cannot be extrapolated. First, homebound individuals are isolated and face restricted healthcare access, often requiring external assistance [3]. Second, global scholars have extensively investigated factors related to facility choices from individual dimensions, facility dimensions, and composite dimensions [18–19]. However, there has been limited research on the association between family-related factors and the primary healthcare preferences of homebound patients. This is particularly important in China where family caregiving is the primary source of care for dependent individuals, who have a greater need for linkage to care resources [18–19]. Third, current research assumes that the preferences for PHC of rural and urban residents are homogeneous, which can lead to inappropriate results [18]. Numerous studies of China’s healthcare system have examined the huge disparities in healthcare utilization that result from rural-urban residence differences [18–20]. The limited regional scope of previous studies on primary healthcare preferences in China and the persistent urban-rural distributional impacts of existing LTC policy disparities warrant investigation of homebound patients’ preferences and associated factors from an urban-rural perspective [20].

Due to gradual spatial confinement or functional limitations, homebound individuals experience changes in their interactions with the environment. Homebound individuals have unique environmental vulnerabilities and a pronounced need for resource connection [21–22]. The Person-Environment Link Model emphasizes the people-related, context-related and linkage-related components that shape the care needed by people living with functional limitations. Our outcome variable (the preference for PHC) and the care needs of people with functional limitations have common properties in that they both indicate the type of care individuals want [21]. The Person-Environment Link model encompasses “people” factors, which encompass individual characteristics such as predisposing, enabling, and needs-based factors [21]. Additionally, it takes into account the “care context,” which encompasses the broader landscape of care provision, including resource availability and community age-friendliness [21]. In our study, we specifically focus on factors related to the context of family caregiving. Furthermore, the model recognizes the significance of “linkage” factors, which encompass various influences on the connection and interaction between individuals and the available care resources [21]. The purpose of this study is to describe the overall proportion of preference for PHC, explore the predictive factors of PHC preferences among both rural and urban homebound residents in China based on the Person-Environment Link model, and provide evidence for policy optimization and tailoring.

## Methods

### Data source and participants

For this study, we utilized the most recent national data (2020) from the China Family Panel Studies (CFPS), which were conducted by the Institute of Social Science Survey at Peking University. The CFPS is a longitudinal survey that began in 2010 and has been conducted every two years since. A multistage probability sampling method with an implicit stratification design was used to select participants [23]. First, based on socioeconomic status in China, primary sampling units were selected from 25 provincial-level administrative regions. Second, a systematic probability proportional to size sampling method was used to sample villages or communities. Third, a cyclical equidistant sampling was conducted to select 28 to 42 households from each village or community, and all family members were invited to participate in the survey. A structured questionnaire was used to collect data on adults through face-to-face interviews.

The first wave of the CFPS contained 42,590 respondents from 14,960 households, while the 2020 survey contained 28,590 respondents. In the 2020 survey, there were 11,930 individuals aged 45 years and older. Inoue and Matsumoto assessed homebound status through a survey question: “Do you leave home without the assistance of others in your daily life?” If the individual answered “no,” they were classified as homebound [2]. In our study, we used two questions to determine homebound individuals. First, we identified 11,770 community residents by measuring “Does this person reside in their home?"; Second, homebound status was determined by the question, “Can you go outdoors independently?” with answers of “yes” or “no.” Respondents missing the homebound response (*n* = 2835) and those selecting “yes” were excluded from the study, and 552 homebound individuals aged 45 years and older were identified finally. After excluding 40 participants with missing values on critical variables - such as primary care preference (*N* = 2), geographic region (*N* = 1), Hukou (*N* = 2), and self-rated health status (*N* = 35), 512 participants were kept in the analysis sample. Participants who reported their current residence location as rural were categorized as rural, while those who reported urban as their current residence location were classified as urban.

### Outcome variable: preference for primary healthcare (PHC)

This study focuses on the preference for PHC, which is determined by whether respondents typically choose PHC as their main source of healthcare [17–18]. This variable was assessed using the question “Where would you usually go to see a doctor?” with response options including: (1) general hospitals, (2) specialty hospitals, (3) community healthcare center/township health center, (4) community health-care station/village clinic, and (5) clinic. Participants who selected PHC institutions (community healthcare center/township health center, community health-care station/village clinic, or clinic) were categorized as having a preference for PHC (coded as “Yes”), while those who chose other healthcare facilities were coded as having no preference for PHC (coded as “No”).

### Explanatory variables

We divided the explanatory variables into three dimensions: people-related factors, care context-related factors, and linkage-related factors, based on the Person-Environment Link (P-E Link) for the care needs of the disabled [21]. This model facilitates a comprehensive understanding of the factors associated with the preference for primary healthcare (PHC) among homebound individuals. We selected a series of candidate variables based on a literature review and the availability of data.

Predisposing factors, enabling resources, and needs-based factors were included in the people-related factors dimension. Predisposing factors consisted of gender (female, male), age (< 65, ≥ 65 years old), marital status (couple, single), and education (lower than primary school, primary school or above). Enabling factors included self-rated socioeconomic status (fair, poor, good), Hukou (agricultural, non-agricultural) [Hukou, is a unique household registration system used as a form of social control to exclude the agricultural Hukou population from access to state-allocated goods, welfare, and entitlements], and geographic region (east, central, and west). Needs-based factors included self-rated health status (poor, fair, good), chronic disease (no, yes), and having received inpatient care in the past year (no, yes). In this study, we assessed disability in Activity of Daily Living (ADL) using the survey questionnaire developed by the Institute of Social Science Survey (ISSS) at Peking University. This questionnaire, derived from the American Activities of Daily Living (ADL) measure, has been refined by the ISSS by condensing the original 14 items into a more streamlined set of 7 items [24–26]. This questionnaire covers 7 activities of daily living: walking, eating, kitchen tasks, using public transportation, shopping, cleaning, and laundry. Participants are presented with a binary response format for each item, indicating whether they can or cannot perform the activity independently. We assigned zero point for independent completion and one point for inability to complete the activity independently for each item, and the level of ADL disability was indicated by cumulative scores across all items. Cognitive function was assessed using a single-item measure that asked participants, “Can you remember the most important things that happened to you in the last week?” The response scale ranged from 1 (remember little) to 5 (remember completely) [26–27]. This single-item measure has been shown to be consistent with the measure of cognitive health by Li et al., which is based on the mean score for memory, word recognition, and mathematical ability [28–29]. Mental health was assessed using the Centre for Epidemiological Studies Depression Scale (CES-D-8), an 8-item scale [30]. The Cronbach’s alpha was 0.707 for the rural sample, 0.768 for the urban sample and 0.733 for the total sample.

Family caregiving context-related factors consisted of receiving family caregiving when ill (no, yes), receiving caregiving from a child in daily routines (no, yes), and receiving financial support from the child (no, yes). Linkage-related factors included the number of close children (0, ≥ 1 person), and having dinner with family members every week (no, yes).

### Statistical analysis

The data analysis was conducted using Stata 15.0. Descriptive statistics were utilized to summarize the characteristics of the home-bound population, employing frequency (percentage) for categorical variables and mean (standard deviation) for continuous variables (Table 1). Chi-squared tests were employed for categorical variables, and t-tests were used for continuous variables to assess differences in the distribution of these variables between the groups that had a preference for PHC and those that did not have a preference for PHC (Table 2). Multivariable logistic models were used to identify potential factors associated with the preference for PHC (Table 3). Multivariable logistic models included characteristics that demonstrated statistical significance (*p* < 0.05) in the Chi-squared tests or t-test results. To test for multicollinearity, the variance inflation factor (VIF) was calculated, where a VIF value greater than 10 suggests severe multicollinearity. The results were reported in terms of odds ratios (OR) and 95% confidence intervals (CI) for each variable.

**Table 1:** Basic characteristics of homebound patients in China

Factors	Total (*n* = 512) Mean/N (SD/%)	Rural (*n* = 332) Mean/N (SD/%)	Urban (*n* = 180) Mean/N (SD/%)
**People-related factor (predisposing factors)**			
**Gender**			
Female	336(65.62)	216(65.06)	120(66.67)
Male	176(34.38)	116(34.94)	60(33.33)
**Age**			
< 65	250(48.83)	168(50.60)	82(45.56)
≥ 65	262(51.17)	164(49.40)	98 (54.44)
**Marital status**			
Couple	427(83.40)	276(83.13)	151(83.89)
Single	85(16.60)	56(16.87)	29(16.11)
**Education**			
Lower than primary school	233(45.51)	136(40.96)	97(53.89)
Primary school or above	279(54.49)	196(59.04)	83(46.11)
**People-related factor (enabling factors)**			
**Self-rated socioeconomic status**			
Fair	266(51.95)	167(50.30)	99(55.00)
Poor	72(14.06)	51(15.36)	21(11.67)
Good	174(33.98)	114(34.34)	60(33.33)
**Hukou**			
Agricultural	407(79.49)	303(91.27)	104(57.78)
Non-agricultural	105(20.51)	29(8.73)	76(42.22)
**Geographic region**			
East	239(46.68)	140(42.17)	99(55.00)
Central	95(18.55)	58(17.47)	37(20.56)
West	178(34.77)	134(40.36)	44 (24.44)
**People-related factor (needs-based factors)**			
**Self-rated health status**			
Poor	265(51.76)	175(52.71)	90(50.00)
Fair	72(14.06)	42(12.65)	30(16.67)
Good	175(34.18)	115(34.64)	60(33.33)
**Chronic disease**			
No	333(65.04)	220(66.27)	113(62.78)
Yes	179(34.96)	112(33.73)	67(37.22)
**Having received inpatient care in the past year**			
No	352(68.75)	232(69.88)	120(66.67)
Yes	160(31.25)	100(30.12)	60(33.33)
**Number of items in the ADL disability**	3.02(1.95)	2.94(1.90)	3.16(2.02)
**CES-D-8** ^**a**^	8.14 (4.86)	8.66 (4.79)	7.18 (4.86)
**Cognitive function**	2.07(1.21)	2.06(1.22)	2.10(1.19)
**Care context-related factor**			
**Receiving family caregiving when ill**			
No	130(25.39)	88(26.51)	42(23.33)
Yes	382(74.61)	244(73.49)	138(76.67)
**Receiving caregiving from child in daily routines**			
No	335(65.43)	218(65.66)	117(65.00)
Yes	177(34.57)	114(34.34)	63(35.00)
**Receiving financial support from their children**			
No	307(59.96)	198(59.64)	109(60.56)
Yes	205(40.04)	134(40.36)	71(39.44)
**Linkage-related factors**			
**The number of close children**			
0 person	234(45.70)	158(47.59)	76(42.22)
≥ 1 person	278(54.30)	174(52.41)	104(57.78)
**Having dinner with family members every week**			
No	57(11.13)	40(12.05)	17(9.44)
Yes	455(88.87)	292(87.95)	163(90.56)

a: CES-D-8 indicates the 8-item Centre for Epidemiological Studies Depression Scale

**Table 2:** Univariate analysis of preference for PHC among rural/urban homebound patients

Factors	Rural (n = 332)		*p*	Urban (n = 180)			*p*
	Yes^a^ Mean/N (SD/%)	No^b^ Mean/N (SD/%)		Yes^a^ Mean/N (SD/%)	No^b^ Mean/N (SD/%)		
**People-related factor (predisposing factors)**							
**Gender**							
Female	130(60.19)	86(39.81)	0.377	51(42.50)	69(57.50)		0.915
Male	64(55.17)	52(44.83)		26(43.33)	34(56.67)		
**Age**							
< 65	105(62.50)	63(37.50)	0.128	38(46.34)	44(53.66)		0.377
≥ 65	89(54.27)	75(45.73)		39(39.80)	59(60.20)		
**Marital status**							
Couple	166(60.14)	110(39.86)	0.160	67(44.37)	84(55.63)		0.324
Single	28(50.00)	28(50.00)		10(34.48)	19(65.52)		
**Education**							
Lower than primary school	72(52.94)	64(47.06)	0.091	35(36.08)	62(63.92)		0.050
Primary school or above	122(62.24)	74(37.76)		42(50.60)	41(49.40)		
**People-related factor (enabling factors)**							
**Self-rated socioeconomic status**							
Fair	93(55.69)	74(44.31)	0.210	46(46.46)	53(53.54)		0.162
Poor	27(52.94)	24(47.06)		5(23.81)	16(76.19)		
Good	74(64.91)	40(35.09)		26(43.33)	34(56.67)		
**Hukou**							
Agricultural	177(58.42)	126(41.58)	0.983	57(54.81)	47(45.19)		< 0.001
Non-agricultural	17(58.62)	12(41.38)		20(26.32)	56(73.68)		
**Geographic region**							
East	82(58.57)	58(41.43)	0.932	34(34.34)	65(65.66)		0.039
Central	35(60.34)	23(39.66)		19(51.35)	18(48.65)		
West	77(57.46)	57(42.54)		24(54.55)	20(45.45)		
**People-related factor (needs-based factors)**							
**Self-rated health status**							
Poor	87(49.71)	88(50.29)	0.002	32(35.56)	58(64.44)		0.098
Fair	26(61.90)	16(38.10)		13(43.33)	17(56.67)		
good	81(70.43)	34(29.57)		32(53.33)	28(46.67)		
**Chronic disease**							
No	140(63.64)	80(36.36)	0.007	54(47.79)	59(52.21)		0.078
Yes	54(48.21)	58(51.79)		23(34.33)	44(65.67)		
**Having received inpatient care in the past year**							
No	159(68.53)	73(31.47)	0.000	65(54.17)	55(45.83)		< 0.001
Yes	35(35.00)	65(65.00)		12(20.00)	48(80.00)		
**Number of items in the ADL disability**	2.77(1.88)	3.18(1.92)	0.058	2.85(1.95)	3.39(2.05)		0.076
**CES-D-8** ^**c**^	8.14 (4.36)	9.39(4.93)	0.018	7.45(5.00)	6.98(4.77)		0.519
**Cognitive function**	2.08(1.19)	2.02(1.26)	0.628	2.06(1.21)	2.13(1.18)		0.695
**Care context-related factor**							
**Receiving family caregiving when ill**							
No	71(80.68)	17(19.32)	< 0.001	28(66.67)	14(33.33)		< 0.001
Yes	123(50.41)	121(49.59)		49(35.51)	89(64.49)		
**Receiving caregiving from child in daily routines**							
No	139(63.76)	79(36.24)	0.006	52(44.44)	65(55.56)		0.538
Yes	55(48.25)	59(51.75)		25(39.68)	38(60.32)		
**Receiving financial support from their children**							
No	127(64.14)	71(35.86)	0.010	43(39.45)	66(60.55)		0.263
Yes	67(50.00)	67(50.00)		34(47.89)	37(52.11)		
**Linkage-related factors**							
**The number of close children**							
0	105(66.46)	53(33.54)	0.005	33(43.42)	43(56.58)		0.881
≥ 1 person	89(51.15)	85(48.85)		44(42.31)	60(57.69)		
**Having dinner with family members every week**							
No	24(60.00)	16(40.00)	0.830	8(47.06)	9(52.94)		0.708
Yes	170(58.22)	122(41.78)		69 (42.33)	94(57.67)		

PHC refers to Primary healthcare; a: "Yes" indicate a preference for PHC; b: "No" indicate a preference against PHC; c: CES-D-8 indicates the 8-item Centre for Epidemiological Studies Depression Scale.

**Table 3:** Logistic regression analysis of predictors of PHC preference among rural/urban areas

Factors	Model 1 Rural		Model 2 Urban	
	OR (95% CI)	P	OR (95% CI)	P
**Education** (reference = Lower than primary school)				
Primary school or above			1.84(0.85, 3.94)	0.117
**Hukou** (reference = agricultural)				
Non-agricultural			0.39(0.18, 0.83)	0.015
**Geographic region** (reference = east)				
Central			2.88(1.14, 7.29)	0.025
West			2.18(0.89, 5.36)	0.087
**Chronic disease** (reference = no)				
Yes	0.95(0.57, 1.57)	0.840		
**Having received inpatient care in the past year** (reference = no)				
Yes	0.33(0.19, 0.59)	< 0.001	0.25(0.11, 0.56)	0.001
**CES-D-8** ^**a**^	0.96(0.91, 1.01)	0.159		
**Receiving family caregiving when ill** (reference = no)				
Yes	0.41(0.21, 0.77)	0.006	0.27(0.11, 0.63)	0.003
**Receiving caregiving from child in daily routines** (reference = no)				
Yes	0.68(0.35, 1.33)	0.272		
**Receiving financial support from their children** (reference = no)				
Yes	1.19 (0.56, 2.50)	0.651		
**The number of close children** (reference = 0)				
≥ 1 person	0.69(0.32, 1.45)	0.332		

The multivariable logistic models included characteristics that demonstrated statistical significance (*p* < 0.05) in the Chi-squared tests or t-test results

PHC refers to Primary healthcare; OR refers to odds ratios; 95% CI refers to 95% confidence intervals; a: CES-D-8 indicates the 8-item Centre for Epidemiological Studies Depression Scale

Model 1 consisted of 332 rural homebound patients. The results of Model 1 were statistically significant, Pseudo R^2^ = 0.1142, *p* < 0.001

Model 2 consisted of 180 urban homebound patients. The results of Model 2 were statistically significant, Pseudo R^2^ = 0.1960, *p* < 0.001

## Results

The selected homebound people are between 45 and 91 years old, with an average age of 64.27. The mean age for rural homebound individuals was 63.67, and for urban homebound individuals, it was 65.35. Table 1 presents the sociodemographic characteristics of the homebound patients in 2020. Out of the 512 homebound patients, approximately 65.62% were female, 51.17% were older people, and 52.93% reported a preference for PHC. Of the 332 homebound patients residing in rural areas, approximately 65.06% were female, 49.40% were older individuals, and 58.43% reported usually choosing PHC services. Among the 180 homebound patients residing in urban areas, approximately 66.67% were female, 54.44% were older individuals, and 42.78% reported a preference for PHC.

Table 2 shows that there were significant unadjusted differences between rural homebound patients who preferred PHC and those who did not, in terms of self-rated health status, chronic disease, having received inpatient care in the past year, CES-D-8, receiving family caregiving when ill, receiving caregiving from a child in daily routines, receiving financial support from a child, and number of close children (*p* < 0.05). Specifically, around 70.43% of rural homebound patients with good self-rated health preferred PHC, and 63.64% of rural patients without chronic diseases preferred PHC. However, only 35.00% of rural patients who have received inpatient care in the past year preferred PHC, and 48.25% of rural patients who received daily care from a child preferred PHC.

Table 2 also revealed significant unadjusted differences between urban homebound patients who preferred PHC and those who did not, in terms of education, Hukou type, geographic region, use of inpatient care in the past year, and receipt of family caregiving when ill (*p* < 0.05). Specifically, only 26.32% of urban homebound patients with non-agricultural Hukou reported a preference for PHC, while 34.34% of urban homebound patients living in the East reported a preference for PHC. Additionally, only 20.00% of urban patients who had received inpatient care in the past year preferred PHC, and only 35.51% of urban patients who received family care when sick preferred PHC.

Table 3 present the results of logistic regression analyses. To ensure there were no serious collinearity problems, we checked the multicollinearity between independent variables. Model 1 had VIF values ranging from 1.09 to 2.39, while Model 2 had VIF values ranging from 1.04 to 1.19. All values were below the conventional threshold value of 10.

Among all rural homebound participants (Table 3, Model 1), individuals who received inpatient care in the past year had 67% lower odds of choosing PHC, compared to those who did not receive such care (OR: 0.33, 95% CI: 0.19–0.59, *p* < 0.001); compared to those who did not receive family caregiving when ill, those who received family caregiving when ill had 59% lower odds of choosing PHC (OR: 0.41, 95% CI: 0.21–0.77, p *=* 0.006).

Among all urban homebound participants (Table 3, Model 2), participants who received inpatient care had 75% lower odds of choosing PHC, compared to those who did not receive inpatient care (OR: 0.25, 95% CI: 0.10–0.56, *p* = 0.001); participants who received family caregiving when ill had 73% lower odds of choosing PHC, compared to those who did not receive such caregiving (OR: 0.27, 95% CI: 0. 11 -0.63, *p* = 0.003); individuals with a non-agricultural background had 59% lower odds of choosing PHC, compared to those with an agricultural background (OR: 0.39, 95% CI: 0.18–0.83, *p* = 0.015); Compared to those living in the eastern part of mainland China, individuals living in the central part of China had 188% higher odds of choosing PHC (OR: 2.88, 95% CI: 1.14–7.29, *p* = 0.025).

## Discussion

As far as we know, this is the first study about patient preferences for PHC among homebound patients in China. Understanding this preference is critical for promoting health policy reform, particularly in light of the government’s 2009 health reform objective of improving PHC utilization. Our study found that only 52.93% of Chinese homebound patients preferred PHC when ill, which was lower than a previous study’s finding of 62.77% [17]. Specifically, 58.43% of rural homebound patients preferred PHC, which was lower than the percentage of rural general patients (72.79%) who preferred PHC. Among urban patients, 42.78% of homebound patients preferred PHC, which was lower than the percentage of urban general patients (51.01%) who preferred PHC [17]. The discrepancy may be due to differences in the study sample. Homebound patients may have worse chronic conditions and unmet care needs due to poorly managed health conditions and delayed access to healthcare, leading to greater demand for specialist medical services [31–33]. Therefore, in addition to unilateral efforts to improve the availability of home visit services for homebound patients from the supply-side perspective, it is crucial to pay more attention to recipients’ attitudes toward seeking healthcare and to determine how best to engage them and encourage them to actively choose PHC when they need to see a doctor.

Regarding the factors associated with preferences for PHC, our research reveals that homebound patients, who had received inpatient care in the past year, in both rural and urban areas, were less likely to opt for PHC. Homebound patients tend to prefer higher-level hospitals with more medical resources, likely due to poor post-discharge self-management behavior and inadequate monitoring after discharge [34–35], resulting in serious health problems, as evidenced by previous studies [34–35]. This finding has significant implications for clinical and policy practices. On one hand, PHC physicians must establish close relationships with homebound patients, as there is an urgent need to foster meaningful connections with this population so that PHC institutions become their first, continuous, comprehensive, and coordinated usual source of medical care for addressing common health problems [36]. On the other hand, given the structural barrier of a lack of human resources in China’s PHC institutions and the capacity of PHC staff to provide home visits, PHC must be improved by optimizing needs-based primary medical care supply strategies (e.g., health education around self-care for minor symptoms or consulting a doctor for potentially more serious symptoms) and developing the role of other PHC team members (e.g., PHC nurse-led telephone advice lines) [37]. Future research should focus on better understanding the health conditions, changes in health conditions, and management needs of post-discharge homebound patients.

Furthermore, this study demonstrates the correlation between urban Hukou and inclination towards PHC. However, the preference for PHC did not show a significant correlation with rural Hukou. Our results indicated that homebound patients living in urban areas and those who had a non-agricultural background were less likely to opt for PHC. These results indicated that Hukou type plays a significant role in exacerbating the disparities between rural and urban areas in terms of PHC preference. The Hukou system, deeply ingrained in Chinese society and originally designed for a dual economy, has resulted in the creation of two distinct welfare systems that exacerbate social hierarchy and differentiation within the country [20]. The Hukou policy in China has a significant bias towards urban areas, providing certain rights and social privileges that result in welfare disparities and social inequity [20]. Hukou restrictions have resulted in inequity across various crucial aspects of social welfare, such as healthcare, housing, education, and other benefits [38]. The Hukou system’s impact on social inequity also extends to homebound patients’ preference for PHC. Additionally, in China, there is a close connection between hukou and the type of basic social health insurance. Some studies have shown that the type of basic social health insurance affects PHC choice [17]. Further investigation is necessary to determine if variations in basic social health insurance coverage resulting from differences in Hukou types are substantial factors in PHC preference.

Our research findings suggest that homebound patients who received caregiving from family members during illness tended to prefer higher-level hospitals over PHC facilities, regardless of whether they resided in rural or urban areas. Several previous studies have revealed that family caregiving is associated with a greater probability of receiving inpatient care, as adult family members assist patients in obtaining more advanced medical treatments [39]. Considering the widespread consensus that family caregivers hold a crucial position in making medical decisions for patients who require care [39–40], it is imperative to take into account the broader circumstances of family caregivers when analyzing the PHC choices of homebound patients. Nevertheless, existing research primarily focuses on susceptible patients within customary care environments and gives scant attention to family caregivers. Based on our findings, it is recommended that policies and measures be put in place to enhance the perspectives of both patients and family caregivers. The lack of attention to family caregivers increases the urgency of recognizing their role in shaping homebound patients’ preferences for PHC. Hence, prioritizing family-based public health strategies over patient-centered approaches is crucial to enhance PHC choices, given the significant role of family caregivers in shaping homebound patients’ preferences for medical care [39–40].

Compared to urban residents living in mainland China’s eastern region, those residing in the central region were less likely to choose PHC. Compared to urban participants living in the eastern part of mainland China, those living in the central part of China were less likely to choose PHC. Some studies have shown that there are significant geographic differences in the distribution of health resources [41]. The eastern urban region of mainland China has a higher level of health resources than the other two regions [41]. In the cities of the eastern region, where people have more choice and easier access to hospitals, PHC is not particularly prevalent [41]. In addition, public trust plays a key role in influencing individuals’ willingness to seek care and adhere to treatment recommendations [42–43]. Many patients in China do not trust the quality of care provided by PHC facilities and prefer to seek care at higher-level hospitals. This lack of trust may be due to perceptions of understaffing and inadequate resources at PHC facilities, as well as a cultural preference for larger hospitals [43]. Increasing public trust in primary health care (PHC) is a key policy objective in China. This objective has the potential to lower the overuse of acute care facilities and enhance the efficiency of the health care system.

There are several limitations to our study that should be noted. Firstly, our definition of “homebound status” only considered patients’ ability to leave their homes independently and did not account for those who may be able to leave but nevertheless remain indoors for extended periods. This may have resulted in an underestimation of the number of homebound patients. Secondly, our data on patients’ preferences for PHC was based on self-reported survey data, which may have been subject to reporting bias if patients misreported their usual healthcare sources. Thirdly, our study did not examine factors such as the GDP level of the city, the number of doctors per thousand people, patients’ healthcare needs, prior experiences with primary healthcare, doctor-patient relationships, and whether the preferred institution of choice provides home visits, which may have influenced their preferences for primary healthcare. Future studies should investigate these factors to gain a better understanding of the impact they have on patients’ preferences for primary healthcare. Fourth, it is a cross-sectional study that focuses on preference for PHC at a single point in time, and the cross-sectional nature of the survey data does not allow us to make causal inferences. Despite these limitations, our study has contributed new findings on the relationship between family caregiving and preferences for PHC among homebound patients in both urban and rural China.

## Conclusion

The proportion of homebound patients who preferred PHC was low in both rural and urban areas. Our study found that homebound patients who had been hospitalized in the previous year and those who had received family care during illness were less likely to prefer PHC, regardless of whether they lived in rural or urban areas. Additionally, among urban homebound patients, those with a non-agricultural background were less likely to prefer PHC. These findings have important implications for policymakers and highlight the need to enhance the quality of regional PHC services, optimize needs-based medical care supply strategies, and develop family-based public health strategies. Future studies could further investigate the impact of the Hukou system on the barriers faced by homebound individuals in their choice of medical institutions.

## Data Availability

The data used in this study were sourced from the China Family Panel Studies (CFPS), a publicly accessible national database. The CFPS survey data is openly available to the public for research purposes. Applicants are required to register and log in to the official CFPS website with a valid email address and submit a request for access to the required database. The datasets analyzed in this study are available in the China Family Panel Studies (CFPS) datasets https://www.isss.pku.edu.cn/cfps/.

## Abbreviations

PHC: Primary healthcare
CFPS: China Family Panel Studies
LCT: Long term care
CES-D-8: The 8-item Centre for Epidemiological Studies Depression Scale
